# Current Biotechnological Advancements on Sustainable Metal and Nutrient Removal

**DOI:** 10.1155/2014/146796

**Published:** 2014-02-16

**Authors:** Li-Yuan Chai, Chong-Jian Tang, Qaisar Mahmood, Xian-Wei Liu

**Affiliations:** ^1^Department of Environmental Engineering, School of Metallurgy and Environment, Central South University, Changsha, Hunan 410083, China; ^2^Department of Environmental Sciences, COMSATS Institute of Information Technology, Abbottabad 22060, Pakistan; ^3^The Biodesign Institute, Arizona State University, Tempe, AZ 85287, USA

The prevalence of various pollutants like nitrogenous, sulphurous, phosphorous compounds, heavy metals, and organic compounds has seriously threatened the sustainability of natural ecosystems and is thus interfering with the natural biogeochemical cycling. Chemical pollution of water bodies can cause serious damage to environmental quality because many of these pollutants are extremely toxic and can in the worst case scenario wipe out entire ecosystems. The world still has not come up with the adequate monitoring system despite the fact that the global chemical pollution continues to grow because of the many “new” pollutants which have entered in use recently. Many thousands of new chemicals are introduced annually in USA alone. The long lasting effects of these pollutants are too varied and diverse and are no more a mystery now. Although nature often has great ability to recover from environmental stresses, the growing demands on water resources and land necessitate the professional application of fundamental knowledge of environmental remediation to ensure the maintenance of environmental quality.

Heavy metal pollution is one of the most important environmental problems today [1]. Various industries produce and discharge wastes containing different heavy metals into the environment, such as mining and smelting of metalliferous, surface finishing industry, energy and fuel production, fertilizer and pesticide industry and application, metallurgy, iron and steel, electroplating, electrolysis, electroosmosis, leatherworking, photography, electric appliance manufacturing, metal surface treating, and aerospace and atomic energy installation. They are widely used in all fields of life, that is, batteries, dyes, alloys, chemical compounds, and pharmaceutical and cosmetic products thus suggesting that the risk of pollution is very high. Thus, metals as resource are becoming a short in supply and also cause serious environmental pollution, threatening human health and ecosystem and also brings about serious environmental pollution, threatening human health and ecosystem. Three kinds of heavy metals are of concern, including toxic metals (such as Hg, Cr, Pb, Cd, and As), precious metals (such as Pd, Pt, Ag, Au, and Ru), and radionuclides (such as U, Th, Ra, and Am). The presence of heavy metal in atmosphere, soil, and water, even in traces, represents a severe risk to all organisms for their long-term toxicological effects. Heavy metal bioaccumulation and biomagnifications in the food chain can be extremely dangerous to human health [1].

Wastewater irrigation is not only used due to scarcity of fresh water but it is also used for protection of environment and for its high nutritive values [2]. Water pollution is a great problem throughout the world and ground water pollution occurs due to disposal of industrial effluents and domestic sewage into watercourses [3]. Wastewater not only provides the supplemental irrigation but also provides the useful nutrients, especially organic matter phosphorous and nitrogen to improve physical properties and fertility of soil [4]. The continuous utilization of raw water for irrigation of leafy and nonleafy vegetables results in metal deposition in soil as well as in undercultivated crops well over the maximum permissible level [5]. Heavy metals enter the human body mainly through two routes, namely, inhalation and ingestion, ingestion being the main route of exposure to these elements in human population. Heavy metals intake by human populations through food chain has been reported in many countries [6]. The health risks of various heavy metals accumulated in these vegetables and crop plants need to be carefully assessed.

PAHs mainly generate from incomplete combustion of fossil fuels [7], oil refinery, and steelmaking [8]. PAHs are widely present in environment and organisms owing to high chemical stability, low water solubility, and high lipid solubility [9]. Food ingestion intake of PAHs increases with the higher trophic level of organisms in food chain through accumulating and transmitting [10]. Research has showed that PAHs have seriously damaged the health of marine mammals on the coast of California [11]. PAHs accumulated effectively in goats, intensifying the biotoxicity [12] and threatening human health, especially for those mainly depending on goat meat. The understanding of the distributions and mother-child transmission of PAHs in various food organisms is desirable and provides useful data to decrease human exposure risk.

The anaerobic bioreactor applies the principles of biotechnology and microbiology, and currently, it has been widely used in the wastewater treatment plants due to high efficiency, low energy use, and green energy generation. Anaerobic digestion is an attractive option for waste treatment practice in which both energy recovery and pollution control can be achieved. The anaerobic processes have become increasingly demanding in the treatment of complex industrial wastewaters, which have the ability to treat high concentrations of organics, and may contain toxic materials or complex substances and even low concentrations of domestic wastewater [13, 14]. Regarding the ability to attain environmental protection and resource preservation, anaerobic treatment process and anaerobic bioreactors have received great attention [13, 15, 16].

Nitrogen pollution has caused severe environmental problems. Since the first discovery of ANAMMOX in the early 1990s, this related technology is developing rapidly. A series of new and outstanding outcomes were achieved in the discovery of new ANAMMOX bacterial species including Brocadia sinica and sulfate-dependent ANAMMOX bacteria (Anammoxoglobus sulfate and *Bacillus benzoevorans*). Since the discovery of the ANAMMOX process [17], it has been regarded as a cost-effective and environment-friendly way to treat wastewater containing high ammonium concentrations [18]. By smart application of ANAMMOX in municipal treatment, wastewater treatment plants could be converted from energy-consuming into energy-producing systems [19]. It is a hot topic in the fields of microbiology and environmental science and engineering due to its merits of effective removal of both ammonium and nitrite under anaerobic conditions with high removal rate, little sludge production, and low operational cost [20–22].

In the recent years, the wastewater treatment strategies have been shifted to one of the most promising methods, that is, biological anaerobic treatment with the adoption of high rate anaerobic systems like up-flow anaerobic sludge blanket (UASB) and other related treatment systems. The outstanding characteristics of high rate anaerobic bioreactor (ABR) include the anaerobic microorganisms capable of aggregation, low operational and maintenance costs, energy recovery in the form of biogas, low energy consumption, and low production of digested sludge [23]. The ABR treated effluents can be employed for irrigation of various crops. However, such type of effluent may be high in chemical oxygen demand (COD), biochemical oxygen demand (BOD), and coliform [24]. As ABR effluents are anticipated to be rich in various nutrients, they can be treated in Constructed Wetlands (CW). CW is a low cost or economical on-site wastewater treatment technology which is not only effective but also aesthetically pleasing. Since 1980, the utilization of the CW for the treatment of a variety of wastewaters has quickly become widespread. The amount of nutrients removed by plants and stored in their tissues is highly relative which depends on the plant type, biomass, and nutrient concentration in tissues [25]. A variety of macrophytes are used in CW and most common are floating macrophytes (i.e., *Lemna* spp. or *Eichhornia crassipes*), submerged macrophytes (i.e., *Elodea canadiensis*), and rooted emergent macrophytes (i.e., *Phragmites australis *and *Typha angustifolia*). The plants roots create conducive environment for the microbial growth and in winter the plant litter acts as insulator. CW are attached growth biological reactors, which tender higher pollutant removal efficiency through physical, chemical, and biological mechanisms. The common removal mechanisms associated with wetlands include sedimentation, coagulation, adsorption, filtration, biological uptake, and microbial transformation [26, 27].

The role of bacteria in remediation of toxic compounds has been documented over the years and would continue to be a dominant technology for the remediation of organic and inorganic compounds [28]. Bacteria with the ability to tolerate, remove, or/and degrade several xenobiotics simultaneously are urgently needed for remediation of sites contaminated with more than one pollutant. Enzymatic reduction of metals to less toxic, nonsoluble lower oxidation states is employed by many bacteria for survival and can be used as a tool for bioremediation of heavy metals.

We hope that readers of CBA will find in this special issue accurate data and updated reviews on the various aspects of the subject. Some useful information has been presented on risk assessment of various pollutants, technological aspects of metal treatments, and strategies of metal recovery.

*Li-Yuan Chai* *Chong-Jian Tang* *Qaisar Mahmood* *Xian-Wei Liu*
